# In Situ Experimental Study on the Behavior of UHPC Composite Orthotropic Steel Bridge Deck

**DOI:** 10.3390/ma13010253

**Published:** 2020-01-06

**Authors:** Li Su, Shilei Wang, Yan Gao, Jianlei Liu, Xudong Shao

**Affiliations:** 1 China Academy of Railway Sciences Corp. Ltd., Beijing 100081, China; su_bjtu@163.com (L.S.); tkygy@126.com (Y.G.); jianleiliu1011@163.com (J.L.); 2 Department of Bridge Engineering, Hunan University, Changsha 410082, China; shaoxd@hnu.edu.cn

**Keywords:** orthotropic steel deck, UHPC, hot spot stress, in situ experiment

## Abstract

A novel ultra high performance concrete (UHPC) layer composite orthotropic steel deck was adopted in the construction of a new bridge in China to improve the fatigue performance of the orthotropic steel deck plate and reduce the disease of surface wearing layer. In situ experiments were conducted to study the UHPC layer’s impact on the behavior of the orthotropic steel deck. The test vehicle loads were applied on the deck plate before and after UHPC layer paving, the stresses where fatigue cracks usually occur and the deflections of critical sections were measured. The test results verified that the UHPC composite steel deck system could significantly reduce the stress of the rib-to-deck connection region and the stress at the bottom toe of rib-to-diaphragm weld. In addition, it slightly influenced the performance of U shape rib, girder web-to-deck and diaphragm cutout.

## 1. Introduction

Orthotropic steel deck (OSD) with its unique advantages, has been popularly used in large and medium span steel bridges. As orthotropic steel deck is the component that bears wheel load directly, the components and their connections of OSD suffer high local stresses creating fatigue cracks during the operation stage. Many investigations indicate that fatigue cracks of OSD mainly occur in the deck plate, the longitudinal weld between deck plate and longitudinal rib, the rib splice joint, and the connection between the rib and the crossbeam. The fatigue cracks of OSD will lead to the breakdown of the wearing surface which affects the safety and comfort of driving. Severe cracks will endanger the overall structure [1,2].

Plenty of experimental and theoretical studies were conducted on the fatigue cracking process, fatigue characteristics as well as failure mechanics of OSD, and the relevant construction technology. Weld penetration rate, structural configuration, measure of maintenance, and reinforcement are proposed to improve the fatigue performance of OSD [3,4,5,6,7,8].

Previous research has indicated that the wearing surface plays a great role in improving fatigue performance of OSD, which effectively reduces fatigue stresses in the steel deck components under repetitive load [9]. The traditional asphalt surfacing has an important influence on the level of stresses of some details in the deck and hence prolonging its fatigue life. However, asphalt is viscoelastic and temperature-dependent, its properties change with age, and the contribution of asphalt pavement is usually not taken into account in fatigue design at present [2]. In addition, during its service period, the traditional asphalt wearing surface of OSD is affected by various diseases including crack, rutting, pit, and so on [10,11]. The causes of the vulnerable problems of the asphalt pavement layer in OSD are very complicated. Insufficient rigidity of steel deck as well as the temperature-dependent property of asphalt could cause the diseases of flexible pavement.

Seim and Ingham [12] put forward the requirements for OSD wearing surfaces. The material should provide the ideal properties such as skid resistance, smooth ride quality, resistant to cracking, durability, and have high bond strength to resist delaminations from shear stresses caused by flexure and by differential temperature expansion and contraction. Therefore, various modified asphalt materials are proposed for OSD, for instance, gussasphalt, mastic asphalt, epoxy asphalt, etc. [13,14,15,16].

On the other hand, high performance concrete was employed to serve as a rigid wearing surface of OSD, which could efficiently improve the whole rigidity of bridge deck system. For instance, reinforced high performance concrete (RHPC) was adopted as the rigid wearing surface of the Caland Bridge in Portland; however, fatigue cracks still occurred in the RHPC layer [17]. A similar phenomenon was also presented in installation using a steel fiber reinforced concrete (SFRC) surface of OSD [18]. The rigid wearing surface could reduce the fatigue stress amplitude of OSD; however, the tensile strength of the material was still not enough to resist the repeated impact from vehicles.

Under this situation, ultra high performance concrete (UHPC) is considered to further improve the tensile strength of the rigid wearing surface of OSD. Hajar et al. [19] adopted ultra-high performance fiber-reinforced concrete (UHPFRC) for repairing the OSD of a bridge, in order to improve the fatigue performance and extend the service lifetime of existing orthotropic slabs. Shao et al. [20] proposed a composite deck system composed of orthotropic steel deck, ultrathin UHPC layer and asphalt pavement. The relevant model experimental study and theoretical calculation were conducted on the behavior and fatigue performance of this composite OSD system [21,22,23,24,25]. The study results showed that the UHPC layer significantly enhanced the rigidity of the OSD system, reduced the stress level of steel deck plate and improved the mechanical behavior of asphalt pavement. In addition, the UHPC layer exhibited superior fatigue performance and durability, which efficiently reduced the lifecycle cost and environmental influence of the bridge. Even though the UHPC composite OSD system has already been applied in some actual bridges, it is necessary to carry out some in situ tests which directly indicate the behavior of the OSD bridges [23,26].

In this paper, an actual bridge with UHPC composite OSD system was selected for an in situ load test. Firstly, the test was conducted at the stage before the deck pavement laying, the vehicle load was directly applied on the steel deck plate. Secondly, the test was conducted at the stage that the UHPC layer has been combined with the deck plate. The stresses where fatigue cracks of OSD mainly occur were tested. This study verified the improvement of the OSD system behavior with UHPC layer, which also helped for evaluating the fatigue life and further optimizing the composite deck components.

## 2. Case Bridge Information

**Figure 1:**
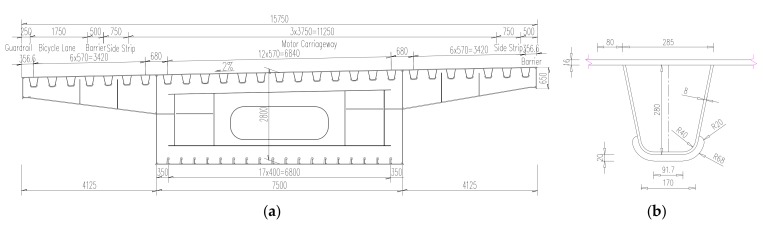
Design drawing of test bridge: (**a**) Cross section of steel box girder; (**b**) Connection detail of the longitudinal and transverse ribs (unit: mm).

The case bridge is comprised of three-span continuous, one-cell welded steel box girders, with the main span of 113 m and side span of 59 m. The bridge, which opened to traffic in 2014, is 15.75 m wide for the deck with 3 lanes for single direction. The bridge adopted an OSD system with a 16 mm steel deck plate. The dimension of the closed U-shaped rib is 285 mm × 280 mm × 8 mm, with transverse interval of 570 mm. Diaphragms are installed every 2.5 m. Cross-section of the steel box girder is shown in Figure 1a and the detail of U-shaped rib is shown in Figure 1b.

**Figure 2:**
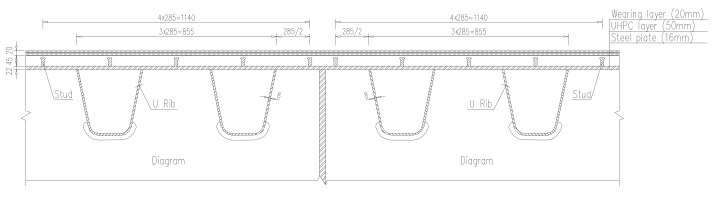
Cross section of composite deck with ultra high performance concrete (UHPC) (unit: mm).

**Figure 3:**
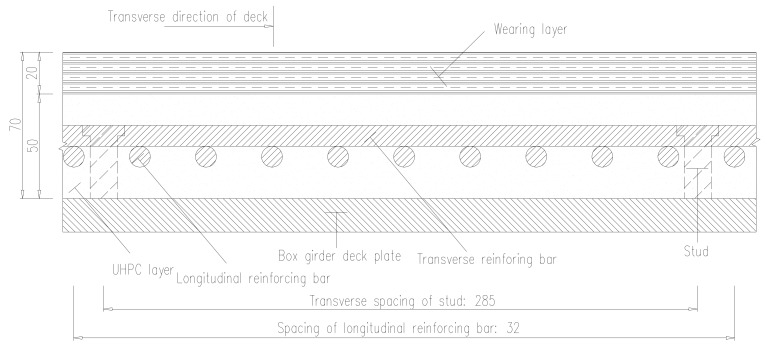
Layout of reinforcing bars and studs (unit: mm).

**Figure 4:**
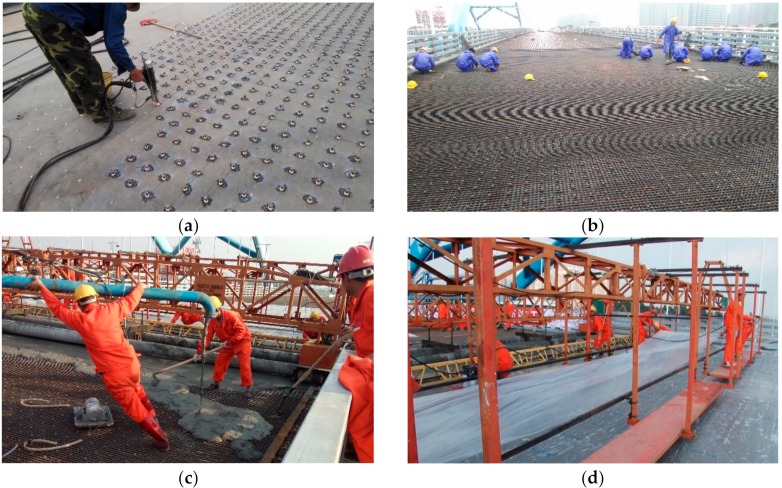
Construction process of UHPC layer of composite OSD: (**a**) Stud welding; (**b**) Reinforcing bar fixing; (**c**) UHPC casting; (**d**) UHPC moist curing.

The composite deck with UHPC is employed to solve the fatigue problem of OSD. Firstly, the studs are welded at the deck plates, and then longitudinal and transverse reinforcing bars are fixed, finally, the UHPC layer is poured. The asphalt concrete wearing layer is paved above the UHPC layer to improve the driving comfort. Cross-section of composite deck with UHPC is shown in Figure 2 and details are shown in Figure 3, Table 1 shows the mechanical properties of all elements of the UHPC composite OSD. The construction process of UHPC composite layer of the case bridge is shown in Figure 4.

## 3. Load Test of the Bridge Deck

### 3.1. Test Content and Arrangement of Testing Points

**Figure 5:**
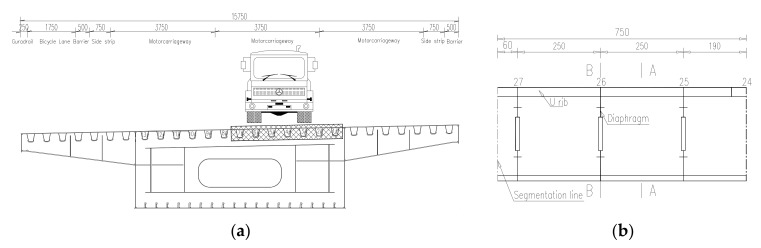
Transverse and longitudinal test zone of instrumentation: (**a**) Transverse test area (unit: mm); (**b**) Longitudinal test sections (unit: cm).

**Figure 6:**
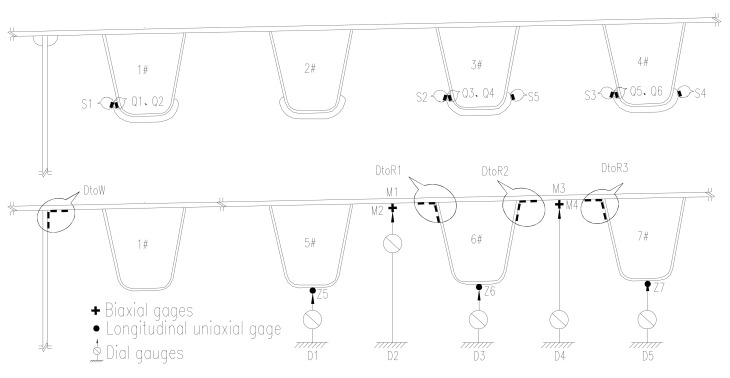
A and B sections layout of instrumentation.

**Figure 7:**
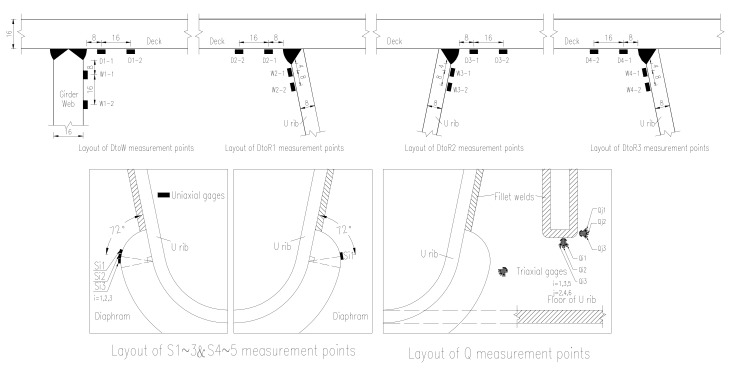
Layout details of strain points.

Based on the behavior of OSD and the lanes plan, the transverse test area is limited to the region of lane (1#–7#U rib) which may suffer heavy vehicle load, the longitudinal test area is comprised of two cross sections (section A and section B as shown in Figure 5). The test contents include the stress of longitudinal weld between deck plate and U-shape longitudinal rib, stress of cut-out of diaphragm, stress of longitudinal weld between deck plate and girder web, stress of longitudinal ribs and stress of steel deck plate, as well as deflections of critical sections. The test area and cross sections are shown in Figure 5; the arrangements of testing points are shown in Figure 6; the details of test points are shown in Figure 7.

### 3.2. Loading Procedures

**Figure 8:**
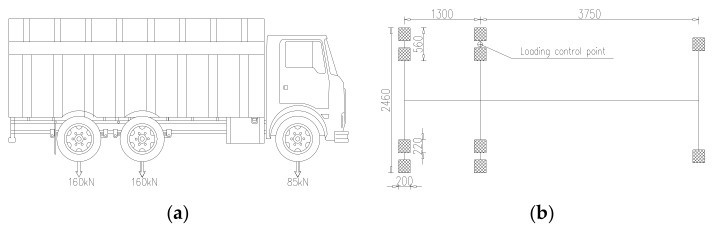
Test vehicle information: (**a**) Load distribution of the test truck; (**b**) Dimension of the wheel/ground contact face. (unit: mm).

A trial-axle truck is selected for static loading, the axle loads of the truck were configured in accordance with the standard of typical overload trucks. Model of the truck and dimension of the wheel/ground contact face are shown in Figure 8.

**Figure 9:**
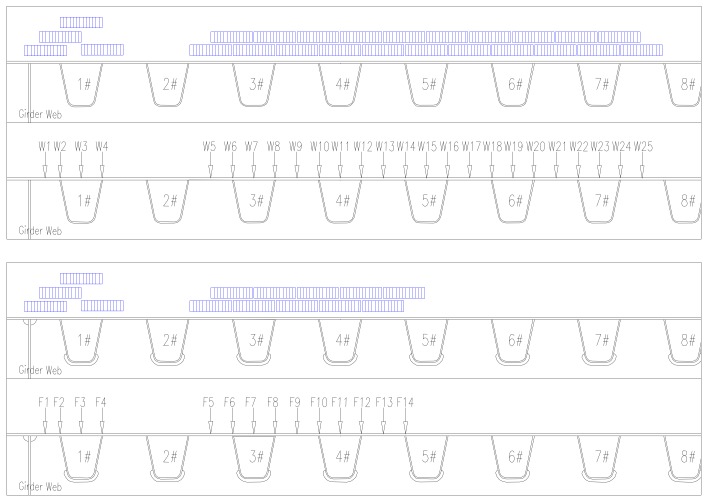
Loading procedures.

The loading procedures are designed based on the influence surface of OSD. There are 25 wheel positions above A cross section, named W1–W25 in turn, 14 wheel positions above B cross section, named F1–F14. The wheel positions are located in the middle of two adjacent longitudinal ribs, top of single longitudinal rib, respectively. The loading point of the truck is controlled by the center line of wheel at single side of the middle axle, as shown in Figure 8b. The test wheel positions are distributed as shown in Figure 9, where the arrows point to the loading positions of loading truck, the corresponding blue rectangles represent the actual loading areas of wheels. The truck stops at every wheel position shown in Figure 9, the corresponding stresses and deflections are measured.

The bridge was tested in two stages. The vehicle loads were applied before and after the UHPC layer combined with deck plate, which were named BC and AC in this paper, respectively. The tests were conducted at night with stable ambient temperature.

## 4. Test Results

### 4.1. Tangential Stresses of Diaphragm Cutout

**Figure 10:**
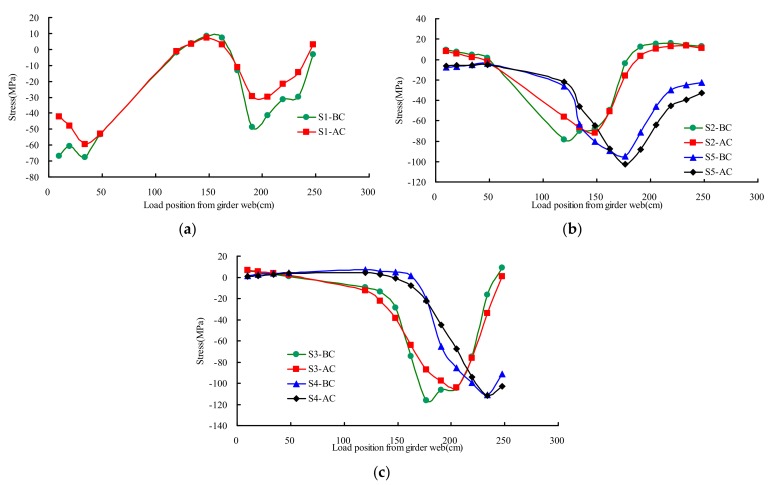
Mean stresses of diaphragm cutout under transverse wheel positions: (**a**) S1; (**b**) S2 and S5; (**c**) S3 and S4.

Figure 10 presented the stresses distribution rule of testing points S1–S5 on different transverse wheel positions. There was no significant change on the stress under test load compared the state of AC and BC. The maximum measured stress of diaphragm cutout was −116 MPa at the state of BC, and the corresponding stress at the state of AC was −112 MPa, the stress of diaphragm cutout slightly decreased 4% after UHPC layer paving.

### 4.2. Principal Stresses of Rib-to-Diaphragm

**Figure 11:**
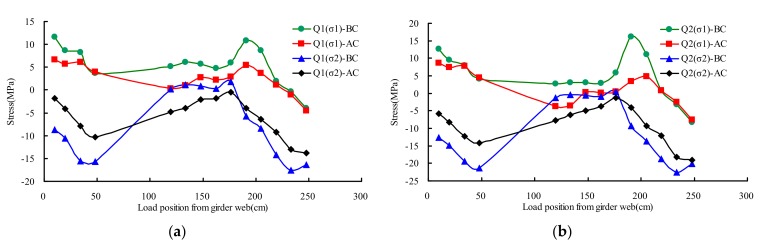

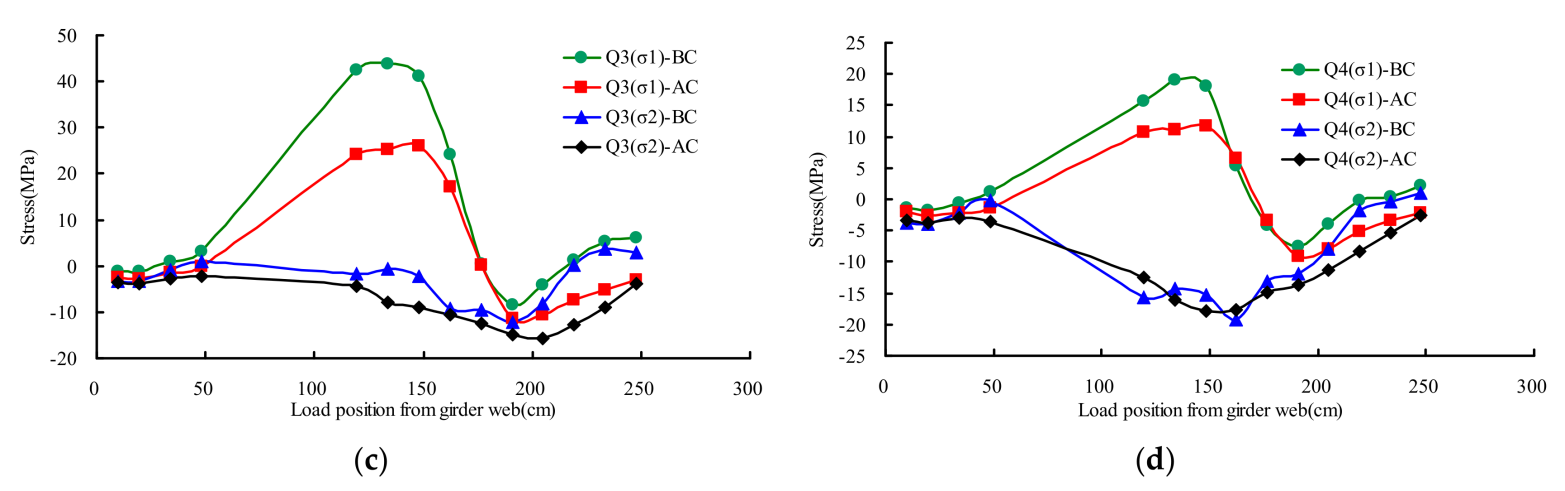
Principal stresses of rib-to-diaphragm under transverse wheel positions: (**a**) Q1; (**b**) Q2; (**c**) Q3; (**d**) Q4.

Figure 11 presented the principal stresses distribution rule of testing point Q1–Q4 on different transverse wheel positions. The principal stresses (σ_1_, σ_2_) and the max shear stress (τ_max_) can be calculated by the Equation (1), based on the measured results obtained from triaxial gages.

(1) $$\begin{array}{l} \sigma_{1}=\frac{E}{2}\left\{\frac{\varepsilon_{0}+\varepsilon_{90}}{1-\mu }+\frac{1}{1+\mu }\sqrt{{\left(\varepsilon_{0}-\varepsilon_{90}\right)}^{2}+{\left[2\varepsilon_{45}-\left(\varepsilon_{0}+\varepsilon_{90}\right)\right]}^{2}}\right\}\\ \sigma_{2}=\frac{E}{2}\left\{\frac{\varepsilon_{0}+\varepsilon_{90}}{1-\mu }-\frac{1}{1+\mu }\sqrt{{\left(\varepsilon_{0}-\varepsilon_{90}\right)}^{2}+{\left[2\varepsilon_{45}-\left(\varepsilon_{0}+\varepsilon_{90}\right)\right]}^{2}}\right\}\\ \tau_{max}\frac{E}{2\left(1+\mu \right)}\sqrt{{\left(\varepsilon_{0}-\varepsilon_{90}\right)}^{2}+{\left[2\varepsilon_{45}-\left(\varepsilon_{0}+\varepsilon_{90}\right)\right]}^{2}} \end{array}$$

Test points Q1 and Q3 are located at the U rib web surface, under the rib-to-diaphragm weld bottom toe. As shown in Figure 11, there was no significant change on the stress in the state of AC and BC. The maximum measured stress (σ_1_) was 44 MPa at the state of BC, and the corresponding stress at the state of AC was 26 MPa. The stress of AC averagely decreased 26% compared with that of BC.

Test points Q2 and Q4 are located at the U rib web surface beside the rib-to-diaphragm weld bottom toe. As shown in Figure 11, there was no significant change on the structural behavior under test load compared to the state of BC. The maximum measured stress (σ_1_) was 19 MPa at the state of BC, and the corresponding stress at the state of AC was 12 MPa. The stress of AC averagely decreased 27% compared with that of BC.

### 4.3. Hotpot Stresses of Rib Web-to-Deck

**Figure 12:**
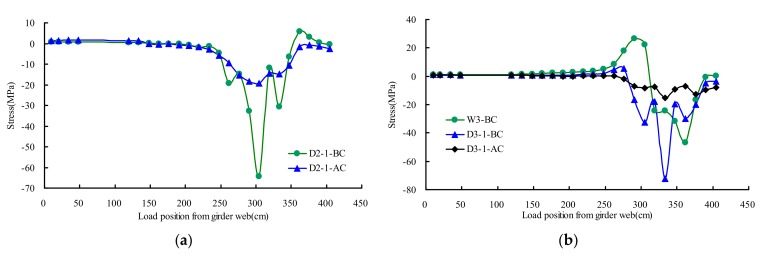

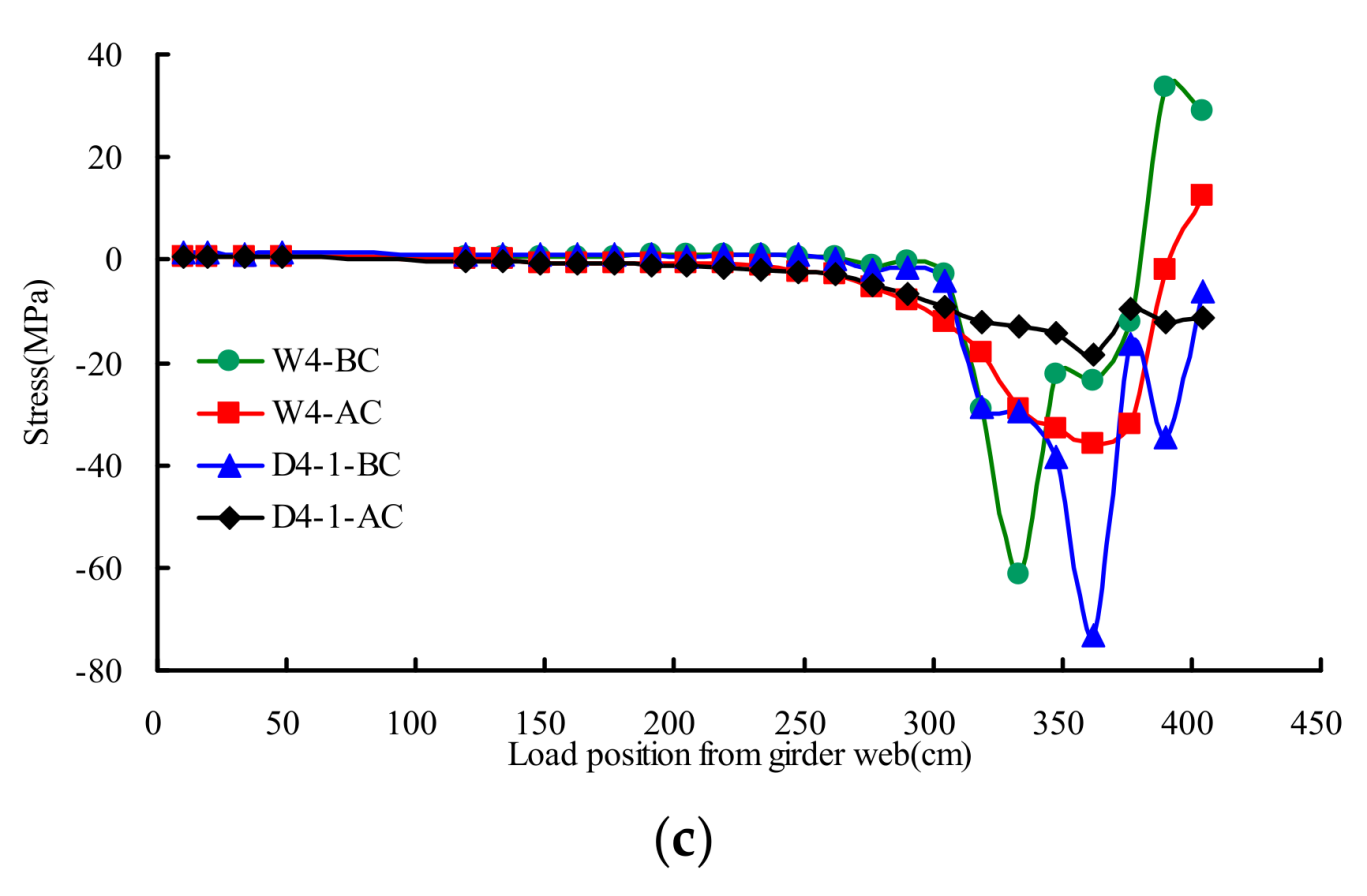
Hotpot stresses of rib web-to-deck under transverse wheel positions: (**a**) D2; (**b**) W3 and D3; (**c**) W4 and D4.

Figure 12 presented the extrapolated hotpot stresses distribution rule of testing point D2–D4 and W2–W4 on different transverse wheel positions. The results showed that the influence line of AC became more gradual than that of BC. The maximum measured stress of the deck weld toe was −73 MPa at the state of BC, and the corresponding stress at the state of AC was −19 MPa. The stress of AC averagely decreased by 75% as compared with that of BC. The maximum measured stress of U rib web weld toe was −61 MPa at the state of BC, and the corresponding stress at the state of AC was −36 MPa. The stress of AC averagely decreased 42% compared with that of BC.

### 4.4. Hotpot Stresses of Girder Web-to-Deck

**Figure 13:**
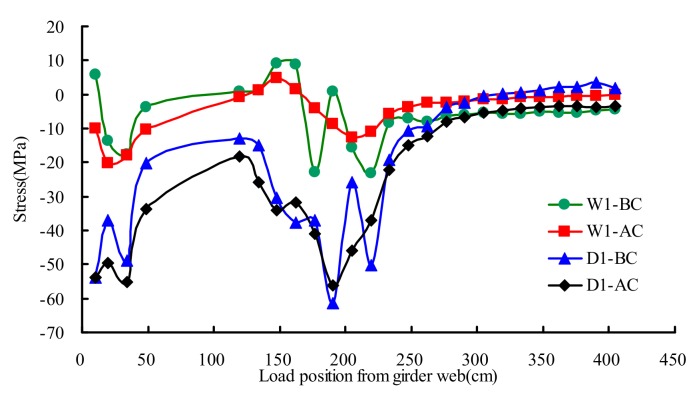
Hotpot stresses of girder web-to-deck under transverse wheel positions.

Figure 13 presented the extrapolated hotpot stresses distribution rule of testing points D1 and W1 on different transverse wheel positions. The results showed that the influence line of AC became more gradual than that of BC. The maximum measured stress of deck weld toe was −61 MPa at the state of BC, and the corresponding stress at the state of AC was −56 MPa. The stress of AC averagely decreased 8% compared with that of BC. The maximum measured stress of girder web weld toe was −23 MPa at the state of BC, and the corresponding stress at the state of AC was −20 MPa. The stress of AC averagely decreased 12% compared with that of BC.

### 4.5. Stresses of Deck Plate

**Figure 14:**
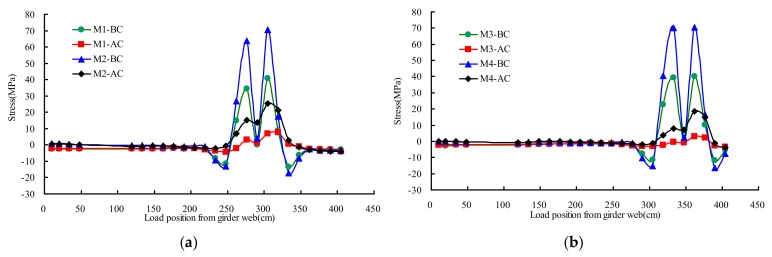
Stresses of deck plate under transverse wheel positions: (**a**) M1 and M2; (**b**) M3 and M4.

Figure 14 described the stresses distribution rule of testing points M1–M4 on different transverse wheel positions. The two orthogonal stresses (σ1, σ2) can be calculated by the Equation (2) based on the measured results obtained from the biaxial gages.

(2) $$\begin{array}{c} \sigma_{1}=\frac{E}{1-\mu^{2}}\left(\varepsilon_{1}+\mu \varepsilon_{2}\right)\\ \sigma_{2}=\frac{E}{1-\mu^{2}}\left(\varepsilon_{2}+\mu \varepsilon_{1}\right) \end{array}$$

The results showed that the influence line of AC became more gradual than that of BC. The transverse stress was more serious than the longitudinal stress. The maximum measured transverse stress of deck was 71 MPa at the state of BC, and the corresponding stress at the state of AC was 25 MPa. The stress of AC averagely decreased 69% compared with that of BC.

### 4.6. Stresses of U-Shape Rib

**Figure 15:**
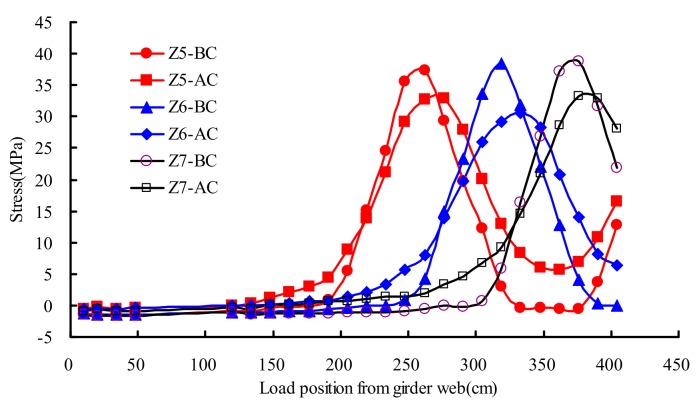
Stresses of U-shape rib under transverse wheel positions.

Figure 15 described the stresses distribution rule of testing points Z5–Z7 on different transverse wheel positions. As shown in Figure 15, there was no significant change on the stress under test load in the state of AC and BC. The maximum measured stress of U rib was 39 MPa at the state of BC, and the corresponding stress at the state of AC was 33 MPa. The stress of AC averagely decreased 16% compared with that of BC.

### 4.7. Deflection of OSD Components

**Figure 16:**
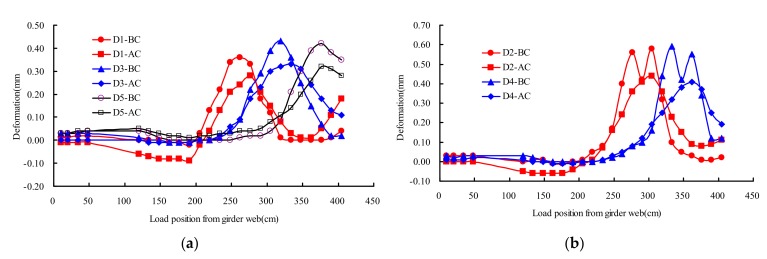
Deflections of deck plate under transverse wheel positions: (**a**) M1 and M2; (**b**) M3 and M4.

Figure 16 described the deflections distribution rule of testing points D1–D5 on different transverse wheel positions. Test points D1, D3, and D5 were located at the U rib, D2 and D4 were located at the deck plate. As shown in Figure 16, there was no significant change on the structural deflection under test load in the state of AC and BC. The maximum measured deflection of U rib and deck plate were 0.43 mm and 0.59 mm at the state of BC, respectively, and the corresponding deflection at the state of AC were 0.33 mm and 0.44 mm, respectively. The deflection of AC averagely decreased 23%–27% compared with that of BC.

## 5. Conclusions

In this paper, an in situ load experiment was designed to study the influence of the UHPC layer on the behavior of a composite OSD system. Under the vehicle load applied, the stresses and deflections of OSD components were tested before and after UHPC layer paving, respectively. Conclusions drawn from experimental results are summarized as follows:

1. The composite deck with UHPC layer significantly reduced the hot spot stress of U rib web-to-deck connection region and the stress of deck plate. The stress of weld toe at U rib web and deck plated decreased 75% and 42%, respectively, and the transverse stress of deck plate decreased 69%. In addition, the UHPC layer significantly improved the integral rigidity of the bridge deck system. The deflection of U rib and deck plate averagely decreased 23% and 27% after UHPC layer paving, respectively;
2. The composite deck with UHPC layer effectively reduced the stress of U rib-to-diaphragm connection region. After UHPC layer paving, the stress of U rib surface, under the rib-to-diaphragm weld bottom toe, averagely decreased 26%. The stress of U rib surface, beside the rib-to-diaphragm weld bottom toe, averagely decreased 27%;
3. The composite deck with UHPC layer slightly reduced the stress of U shape rib, girder web-to-deck and diaphragm cutout.

The fatigue problem of OSD system is a traditional task in steel bridge study. The modified deck pavement of OSD is a hot topic in recent years, because it distinctly improves the fatigue performance of OSD system. The UHPC composite OSD system is a novel structural configuration. In earlier laboratory tests and finite element analysis, the UHPC composite OSD system was verified to significantly reduce the stresses where fatigue cracks mainly occurred, compared to OSD system with asphalt pavement [20,21]. The case bridge in this paper, was the first application of UHPC composite OSD with closed stiffening rib in China. The practical engineering application is different from laboratory tests because the field construction condition is complex, including construction quality, curing condition, environmental condition and so on. In this study, the actual improvement of mechanical behavior of OSD contributed from UHPC composite layer was tested under a specific load, before and after UHPC layer paved. The test results also verified that the behavior of OSD system was improved by the composite UHPC layer. After that, the UHPC composite OSD system was widely used in other steel bridges in China, including a suspension bridge with a span of 1480 meters [22].

**Figure 17:**
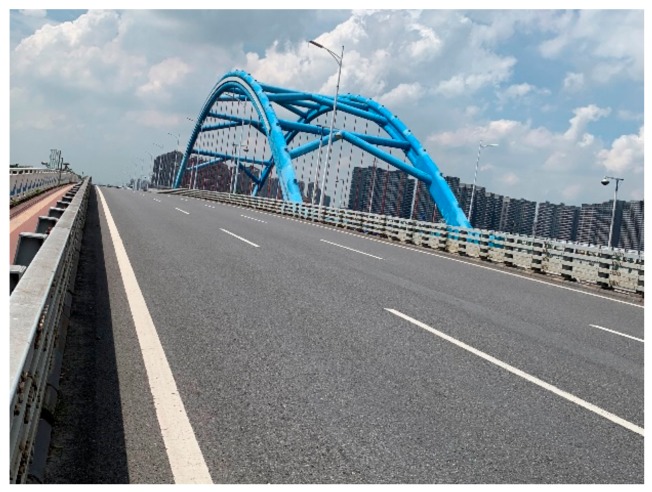
Current situation of the case bridge.

In the conventional OSD system, fatigue cracks chronically occur at the U rib web-to-deck connection region, which is always to the disadvantage of the completeness of pavement and traffic security. Additionally, it is difficult for the bridge maintenance department to rapidly repair these kinds of diseases. The UHPC composite layer could significantly reduce the stresses and prolong the fatigue life of U rib web-to-deck connection region. During five years of operation, the UHPC layer keeps the surface wearing of the case bridge in a good condition, as shown in Figure 17.

In addition, UHPC composite layer slightly reduced the stress of U shape rib, girder web-to-deck and diaphragm cut-out. Further studies are called for better welding technology to improve the quality of weld joint, and more appropriate configuration of diaphragm cut-out to avoid stress concentration.

## Figures and Tables

**Table 1:** Mechanical properties of the elements of the UHPC composite orthotropic steel deck (OSD).

Elements	Density (kg/m^3^)	Elastic Modulus (MPa)	Poisson’s Ratio	Tensile Strength (MPa)	Compressive Strength (MPa)
steel deck plate	7850	2.06 × 10^5^	0.30	345	-
reinforcing bar	7850	2.06 × 10^5^	0.30	400	-
stud	7850	2.06 × 10^5^	0.30	375	-
UHPC	2700	4.26 × 10^4^	0.2	7.89	129.1
asphalt wearing surface	2430	1.00 × 10^3^	0.30	1	-
